# Increased Risk of Colon Cancer in Men in the Pre-Diabetes Phase

**DOI:** 10.1371/journal.pone.0070426

**Published:** 2013-08-02

**Authors:** Adedayo A. Onitilo, Richard L. Berg, Jessica M. Engel, Ingrid Glurich, Rachel V. Stankowski, Gail Williams, Suhail A. Doi

**Affiliations:** 1 Department of Hematology/Oncology, Marshfield Clinic Weston Center, Weston, Wisconsin, United States of America; 2 School of Population Health, University of Queensland, Brisbane, Australia; 3 Marshfield Clinic Research Foundation, Marshfield, Wisconsin, United States of America; 4 Department of Hematology/Oncology, Marshfield Clinic Cancer Care, Stevens Point, Wisconsin, United States of America; University of Porto, Portugal

## Abstract

**Background:**

Historically, studies exploring the association between type 2 diabetes mellitus (DM) and cancer lack accurate definition of date of DM onset, limiting temporal analyses. We examined the temporal relationship between colon cancer risk and DM using an electronic algorithm and clinical, administrative, and laboratory data to pinpoint date of DM onset.

**Methods:**

Subjects diagnosed with DM (N = 11,236) between January 1, 1995 and December 31, 2009 were identified and matched at a 5∶1 ratio with 54 365 non-diabetic subjects by age, gender, smoking history, residence, and diagnosis reference date. Colon cancer incidence relative to the reference date was used to develop Cox regression models adjusted for matching variables, body mass index, insurance status, and comorbidities. Primary outcomes measures included hazard ratio (HR) and number needed to be exposed for one additional person to be harmed (NNEH).

**Results:**

The adjusted HR for colon cancer in men before DM onset was 1.28 (95% CI 1.04–1.58, *P* = 0.0223) and the NNEH decreased with time, reaching 263 at DM onset. No such difference was observed in women. After DM onset, DM did not appear to alter colon cancer risk in either gender.

**Conclusions:**

Colon cancer risk is increased in diabetic men, but not women, before DM onset. DM did not alter colon cancer risk in men or women after clinical onset. In pre-diabetic men, colon cancer risk increased as time to DM onset decreased, suggesting that the effects of the pre-diabetes phase on colon cancer risk in men are cumulative.

## Introduction

**Figure 1:**
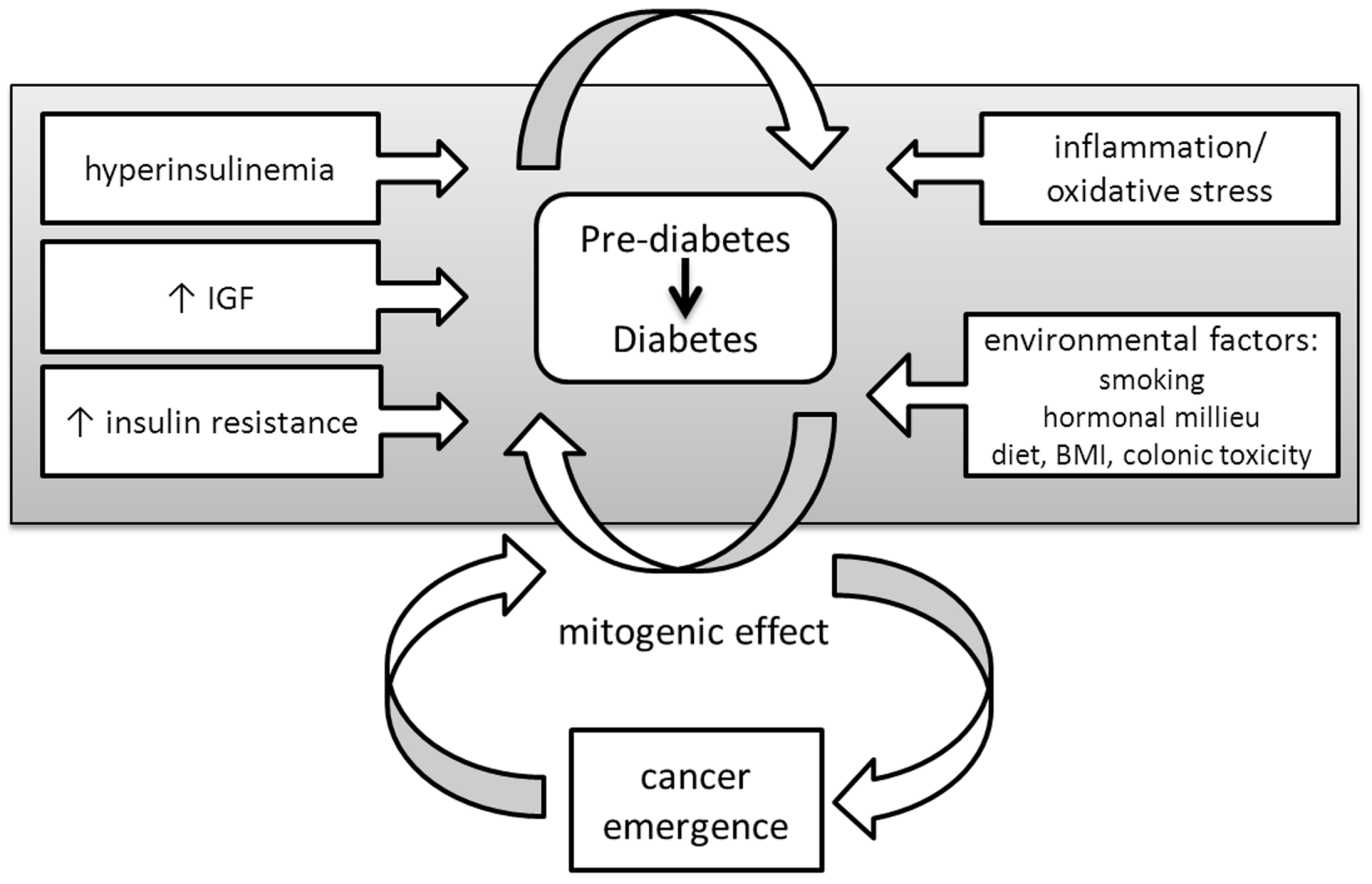
Progression of type 2 diabetes mellitus (DM) from pre-diabetes to clinical onset. DM is a progressive disease in which several important physiological changes occur prior to the onset of overt clinical disease. Several changes have potential mitogenic activity and may stimulate underlying carcinogenic processes.

Pre-diabetes is characterized by chronic inflammation, hyperinsulinemia, increased insulin-like growth factor (IGF) levels, and insulin resistance [1]. These processes are thought to facilitate colonic tumorigenesis through insulin and IGF-stimulated proliferation and increased environmental toxicity resulting from reduced bowel activity and increased fecal bile acid concentrations (Figure 1) [2]. Physiological changes begin long before clinical DM onset, with an average pre-diabetes duration of approximately 9–10 years [3], yet until now, no study has examined colon cancer risk in the pre-diabetes phase. However, data regarding the time period post-diabetes onset does exist with a recent meta-analysis by Luo et al. [4] reporting a relative risk (RR) for colorectal cancer in diabetic compared to non-diabetic subjects of 1.28 (95% CI 1.19–1.39), with little heterogeneity among the 24 included studies.

Gender differences further complicate the relationship between DM and colon cancer with some meta-analyses reporting either a stronger association in men than women [4], [5], a similar RR for diabetic men and women [6] or even a greater risk for colorectal cancer in women post-DM onset [7]. The potential for differences in colon cancer risk in the pre-diabetes phase and the role of gender during that time is, therefore, conflicting and necessitates further study. Here we address some of the limitations of previous studies to report on the association between colon cancer and DM in the prediabetes and post-diabetes phases separately. Meticulous attention was devoted to ascertainment of the date of DM onset and the temporal trends in colon cancer risk were also examined by gender.

## Methods

### Ethics Statement

Study approval and a waiver of informed consent were granted by the Marshfield Clinic Institutional Review Board.

### Study Design

Colon cancer risk before and after DM diagnosis was assessed retrospectively in a matched cohort study at Marshfield Clinic, a large multi-specialty group practice healthcare system in north-central Wisconsin. Data were collected electronically from Marshfield Clinic’s comprehensive electronic medical record (EMR) [8] and cancer registry. The study period included January 1, 1995 through December 31, 2009. Potential subjects had to be 30-years-old by the end of the study period with no diabetes-related diagnoses before the study period.

### Subject Selection and Matching

**Figure 2:**
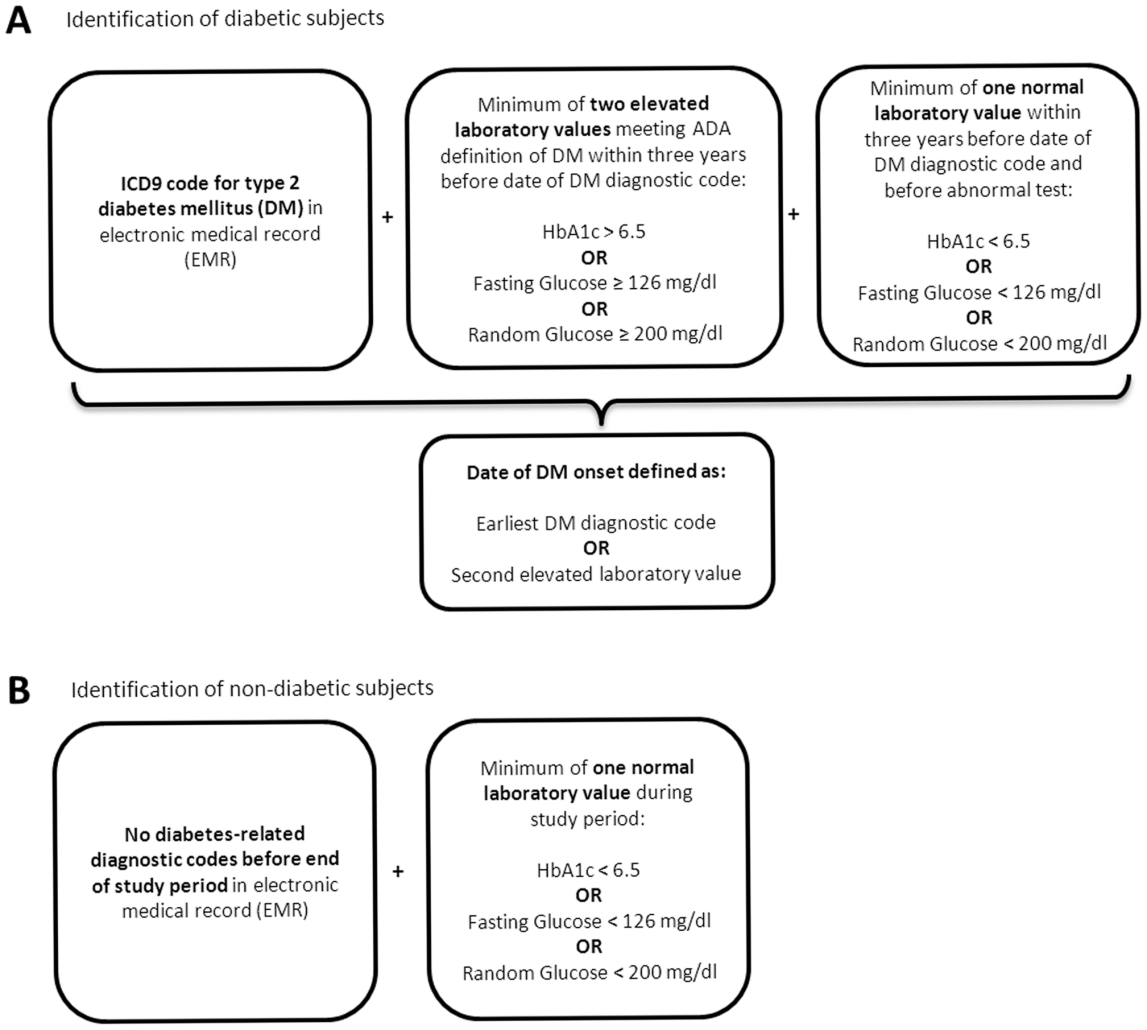
Definition of type 2 diabetes mellitus (DM). (A) The algorithm for defining patients with DM relied on both electronic diagnostic codes and laboratory results. (B) The algorithm for defining patients without DM also relied on electronic diagnostic codes and laboratory results. Normal and elevated laboratory results for HbA1c and glucose are based on American Diabetes Association guidelines (ADA) [12].

Potential diabetic subjects were those with one or more diabetes-related diagnoses during the study period. Those with no diabetes-related diagnoses before the end of the study period made up the pool of potential subjects for the non-diabetic cohort. DM was defined using diagnostic codes and laboratory results (Figure 2) as defined by the American Diabetes Association (ADA) [9]. Subjects with any other type of diabetes (e.g., type 1, gestational) or treated with diabetes medications 30 days or more before diagnosis were excluded. DM onset was defined as the earliest of first diagnostic code or second high laboratory value. Non-diabetic subjects were also verified by laboratory values and clinical data (Figure 2). Potential non-diabetic subjects with no normal glucose or HbA1c tests and those treated with diabetes medications during the study period were excluded. Subjects were required to have received sufficient care through the Marshfield Clinic system, so that diagnosis dates for DM and/or colon cancer could be determined with reasonable accuracy. All subjects were required to have at least one non-DM diagnosis or electronic code documenting a well-visit from a Marshfield Clinic provider in at least one of the three calendar years before the reference date. Observation times were censored before any large gap in the EMR of four or more consecutive calendar years.

**Figure 3:**
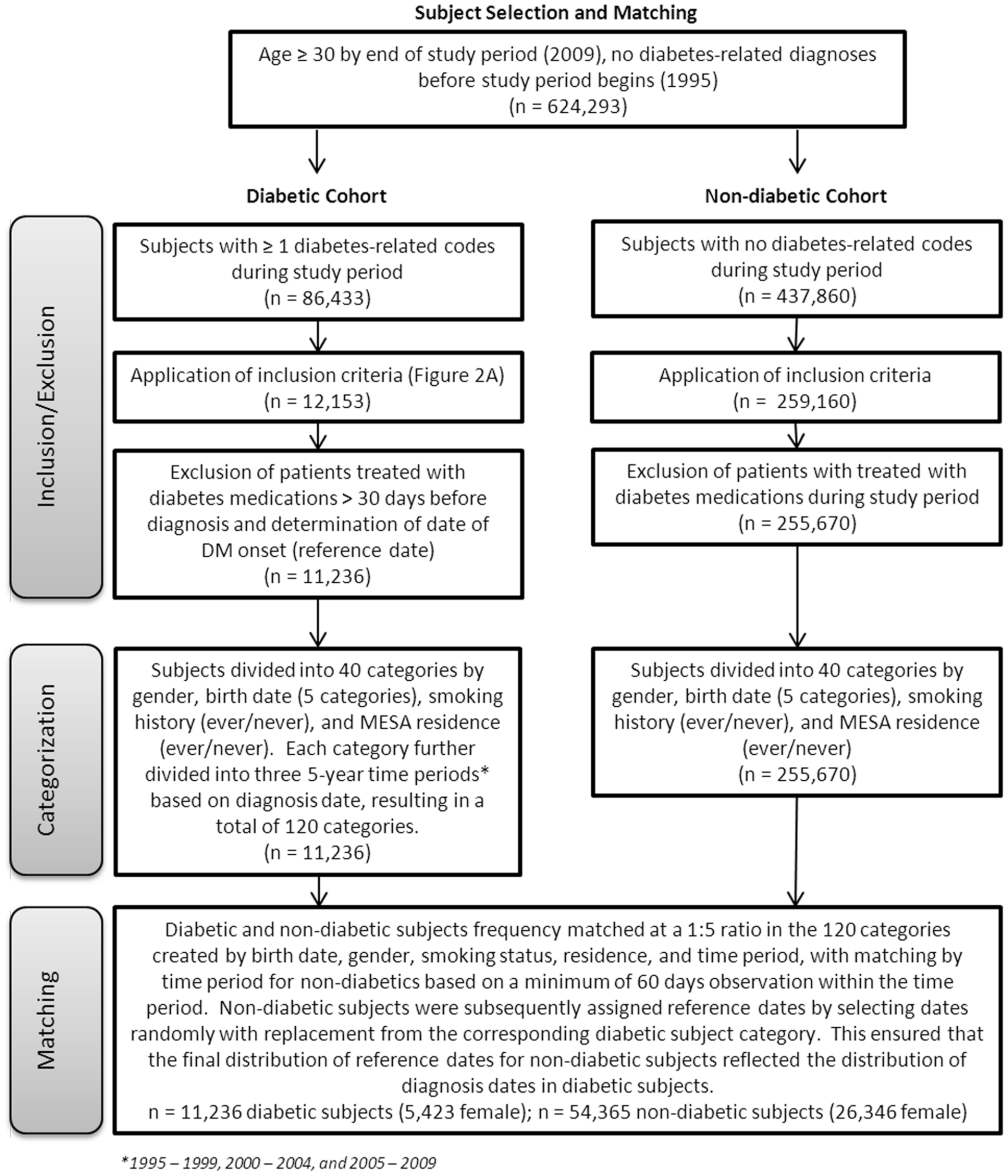
Subject selection and matching. Study subjects were selected carefully based on whether or not they developed type 2 diabetes mellitus (DM) in the time period from January 1, 1995 through December 31, 2009.

Frequency matching of diabetic and non-diabetic subjects at a ratio of 1∶5 was performed as described in Figure 3. Matching on date of diabetes diagnosis was done by dividing these dates into three 5-year reference periods (1995–1999, 2000–2004, or 2005–2009) and assigning potential non-diabetic subjects to one of these time periods if their observation time in the EMR for that period was at least 60 days. Specific reference dates were assigned to non-diabetic subjects by randomly sampling (with replacement) diagnosis dates from the diabetic subjects in the same matching category. This ensured a similar distribution of reference dates in non-diabetic subjects and diagnosis dates in diabetic subjects. Both reference/diagnosis dates are referred to as ‘reference dates’ subsequently and fell within the 15-year study period. However, follow-up extended through 2011 and observation before the reference date went back as far as the patient’s history in the EMR.

### Cancer Diagnosis, Comorbidities, and Clinical Risk Factors

Diagnoses of colon cancer were captured by International Classification of Diseases, Ninth Revision (ICD-9) code. Selected covariates with the potential to impact on colon cancer risk were also examined including comorbidities, clinical risk factors, and cancer treatments. Selected comorbidities, listed in Table 1, were summarized using a modified Charlson score, which excluded cancer and DM. Comorbid diagnoses were validated with at least two diagnostic codes in the EMR. BMI, smoking history (ever/never), and insurance status (yes/no) at reference date, as well as frequency of healthcare encounters with the Marshfield Clinic system in the 2 years before and after the reference date were also abstracted from the EMR.

**Table 1:** Descriptive characteristics and matching variables in diabetic and non-diabetic subjects.

Variables	Diabetic (N = 11 236)	Non-diabetic (N = 54 365)
	N (%)	N (%)
Gender		
Male	5813 (51.7)	28 019 (51.5)
Female	5423 (48.3)	26 346 (48.5)
Mean age (years) (IQR)	62.9 (53–73)	63.2 (53–72)
Age group		
30–49 years	1940 (17.3)	9 760 (18.0)
50–59 years	2681 (23.9)	12 832 (23.6)
60–69 years	3110 (27.7)	14 294 (26.3)
70–79 years	2409 (21.4)	10 711 (19.7)
≥80 years	1096 (9.8)	6 768 (12.4)
Birth year		
1929 and prior	2835 (25.2)	13 856 (25.5)
1930–1939	2779 (24.7)	13 348 (24.6)
1940–1949	2741 (24.4)	13 459 (24.8)
1950–1959	1954 (17.4)	9580 (17.6)
1960 and later	927 (8.3)	4122 (7.6)
Smoking status		
Ever	7579 (67.5)	36 427 (67.0)
Never	3657 (32.5)	17 938 (33.0)
DM diagnosis period		
1995–1999	2486 (22.1)	12 123 (22.3)
2000–2004	4657 (41.4)	22 581 (41.5)
2005–2009	4093 (36.4)	19 661 (36.2)
MESA residency		
No	9036 (80.4)	43 871 (80.7)
Yes	2200 (19.6)	10 494 (19.3)
Mean BMI (kg/m^2^) (IQR)	33.4 (28–37)	28.9 (26–39)
Have insurance	8881 (79.0)	40 852 (75.1)
Visit frequency 2 years before DM		
0–5	2285 (20.3)	15 848 (29.2)
6–10	2367 (21.1)	13 093 (24.1)
11–20	3146 (28.0)	1411 (26.0)
>20	3438 (30.6)	11 308 (20.8)
Visit frequency 2 years after DM		
0–5	1175 (10.5)	21 396 (39.4)
6–10	1523 (13.6)	10 286 (18.9)
11–20	3223 (28.7)	11 492 (21.1)
>20	5315 (47.3)	11 191 (20.6)
Mean observation time (IQR)		
Years Before DM onset	16.6 (6.1–26.1)	16.3 (5.7–26.2)
Years After DM onset	7.4 (4.4–10.0)	6.1 (2.8–8.8)
Comorbidities		
Myocardial infarction	208 (1.9)	609 (1.1)
Coronary heart disease	590 (5.3)	1380 (2.5)
Peripheral vascular disease	272 (2.4)	984 (1.8)
Cardiovascular disease	351 (3.1)	1290 (2.4)
Chronic pulmonary disease	1069 (9.5)	3106 (5.7)
Rheumatic heart disease	200 (1.8)	999 (1.8)
Renal disease	207 (1.8)	713 (1.3)
Cancer Treatment^a^	N = 85	N = 437
Chemotherapy		
No	100 (0.9)	411 (0.8)
Yes	43 (0.4)	231 (0.4)
Radiation		
No	135 (1.2)	581 (1.1)
Yes	8 (0.1)	61 (0.1)

***Abbreviations***: DM, type 2 diabetes mellitus; IQR, interquartile range; MESA, Marshfield Epidemiologic Study Area; BMI, body mass index.

a Treatment data for colon cancer, only available for subjects in cancer registry, N as indicated.

### Statistical Analysis

Risk analyses were performed separately for the time periods before and after the reference date. In the pre-diabetes phase, subject records were examined for colon cancer diagnoses before the reference date and cumulative incidence was plotted starting 15 years before the reference date (year −15). Inclusion of subjects who developed colon cancer before the 15-year period preceding the reference date resulted in a baseline risk at year −15 greater than zero. Subjects with colon cancer diagnoses before the reference date were excluded from analysis of colon cancer risk after the reference date such that cumulative colon cancer incidence started over at zero. After the reference date, records for all subjects with no past history of colon cancer were examined starting at the reference date (year 0) to 15 years after the reference date (year 15).

Baseline subject characteristics at the reference date were summarized using standard descriptive statistics. Colon cancer incidence before and after the reference date was calculated using person-time based on age of participants at the reference date. Primary study outcomes included hazard ratios (HRs) calculated using proportional hazards regression modeling by DM status and adjusted for relevant covariates. Models were analyzed separately for each time period. Results were reported as HR with 95% confidence interval (CI). The following formula was used to calculate the number needed to be exposed to DM for one additional person to be harmed (i.e. develop cancer) (NNEH): 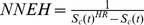, where 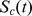
is the probability of a non-diabetic subject being alive and cancer-free at specified time *t*, relative to the reference date [10]. Analyses were conducted with SAS® version 9.2 statistical software.

## Results

A total of 624 293 potential study subjects were identified, of which 86 433 had one or more diabetes-related code during the study period and 437 860 did not. Application of clinical and laboratory criteria resulted in a diabetic cohort containing 11 236 diabetic subjects. Notably, application of laboratory parameters to potential diabetic subjects reduced the numbers by approximately 70% (Figure 3). Diabetic subjects were matched to 54 365 non-diabetic subjects resulting in a final ratio of 4.83 non-diabetic subjects per diabetic subject. Figure 3 summarizes subject selection and matching.

Baseline subject characteristics at the reference date are summarized in Table 1. The cohorts were well-balanced across the matching variables. BMI was higher in those with than without DM, and more healthcare encounters were noted during the 2 years before the reference date among those with DM.

**Figure 4:**
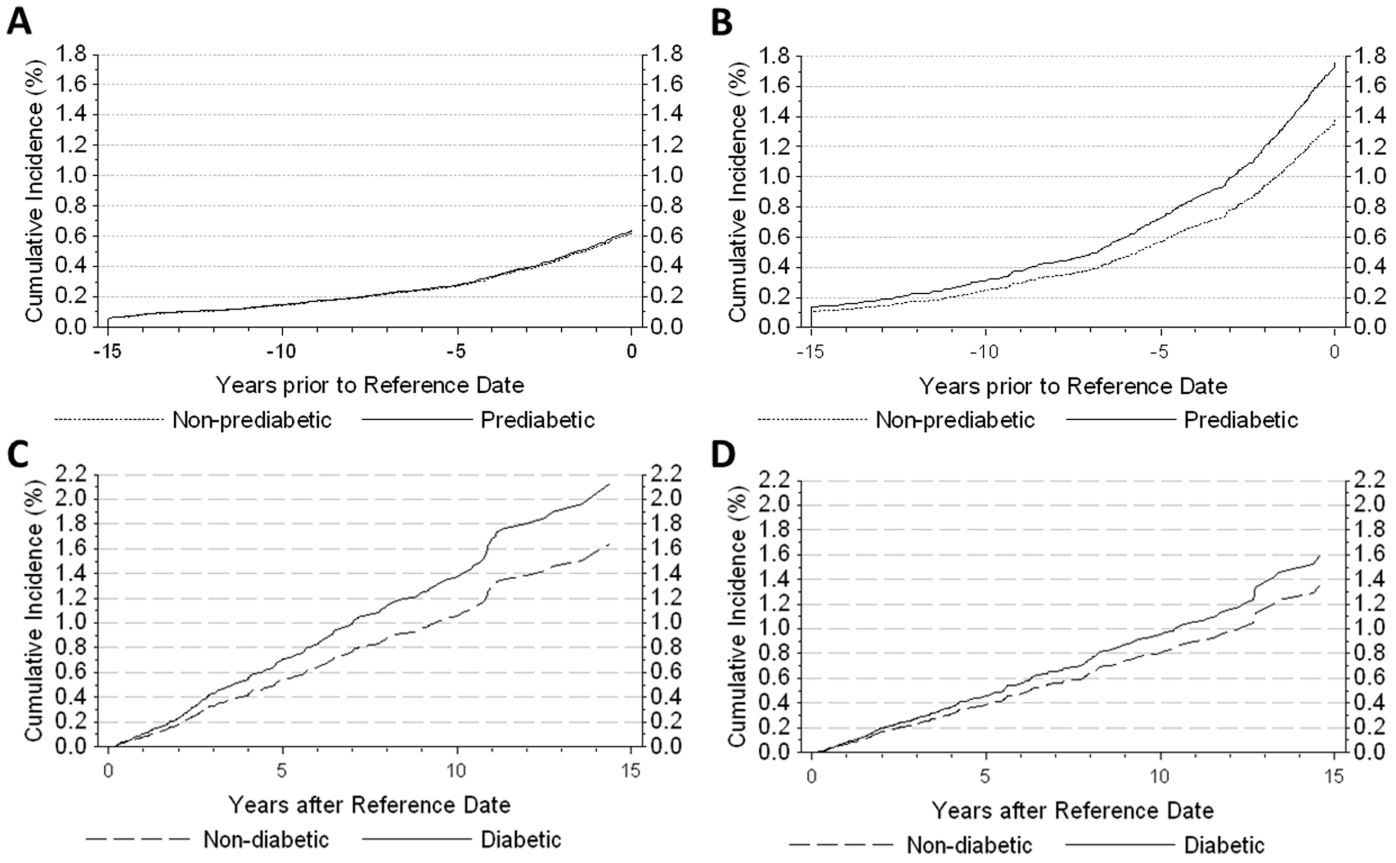
Cancer incidence before and after onset of type 2 diabetes mellitus (DM). Cumulative incidence of colon cancer in women (A) and men (B) before DM onset and women (C) and men (D) after DM onset. Diabetic subjects are indicated by the solid line and non-diabetic subjects are indicated by the dashed line.

Crude colon cancer incidence before the reference date was 27.8 per 100 000 person-years in diabetic subjects and 24.7 per 100 000 person-years among non-diabetic subjects. Gender differences in colon cancer risk were observed in the pre-diabetes phase (Figure 4A and 4B). In women, colon cancer risk was similar in diabetic and non-diabetic subjects before the reference date (Table 2). In men, colon cancer risk before DM diagnosis was increased compared to non-diabetic subjects with an HR of 1.28 (95% CI 1.04–1.58, *P* = 0.0223) (Table 3), and the NNEH decreased over time reaching 263 at DM onset (Table 4).

**Table 2:** Colon cancer risk in women before and after DM onset.

	Before DM Onset			After DM Onset		
	N (%)^a^	HR (95% CI)	*P*-value	N (%)^a^	HR (95% CI)	*P*-value
Diabetes status						
Yes	79 (1.5)	1.03 (0.80–1.32)	0.8392	50 (1.1)	1.30 (0.94–1.81)	0.1162
No	378 (1.4)	1.00 (ref)		170 (0.76)	1.00 (ref)	
Smoking						
Yes	271 (1.5)	1.00 (ref)		125 (0.83)	1.00 (ref)	
No	186 (1.4)	0.88 (0.72–1.06)	0.1763	95 (0.80)	0.65 (0.50–0.86)	0.0021
MESA residency						
Yes	89 (1.4)	1.00 (ref)		46 (0.89)	1.00 (ref)	
No	368 (1.4)	0.92 (0.73–1.16)	0.4877	174 (0.80)	0.95 (0.68–1.32)	0.7456
Birth year						
1929 and prior	262 (2.9)	21.2 (7.82–57.4)	<0.0001	117 (1.7)	40.8 (5.63–295.8)	0.0002
1930–1939	126 (1.7)	11.2 (4.14–30.5)	<0.0001	57 (0.92)	17.3 (2.38–125.4)	0.0048
1940–1949	51 (0.68)	4.24 (1.53–11.7)	0.0055	37 (0.57)	10.4 (1.43–76.2)	0.0209
1950–1959	14 (0.26)	1.55 (0.51–4.69)	0.4433	8 (0.16)	3.05 (0.38–24.4)	0.2934
1960 and later	4 (0.17)	1.00 (ref)		1 (0.05)	1.00 (ref)	
DM diagnosis period						
1995–1999	135 (1.9)	0.71 (0.55–0.91)	0.0079	84 (1.4)	0.46 (0.31–0.70)	0.0003
2000–2004	163 (1.2)	0.66 (0.53–0.82)	0.0002	84 (0.75)	0.56 (0.39–0.81)	0.0018
2005–2009	159 (1.4)	1.00 (ref)		52 (0.54)	1.00 (ref)	
BMI (kg/m^2^)						
<25	92 (1.5)	1.00 (ref)		39 (0.75)	1.00 (ref)	
25–29.9	259 (1.6)	0.89 (0.70–1.20)	0.3793	134 (1.0)	1.06 (0.73–1.54)	0.7471
≥30	106 (1.1)	0.95 (0.71–1.27)	0.7402	47 (0.57)	0.77 (0.50–1.20)	0.2523
Insurance						
Yes	383 (1.6)	1.00 (ref)		177 (0.86)	1.00 (ref)	
No	74 (1.0)	0.87 (0.66–1.14)	0.3097	43 (0.68)	0.80 (0.54–1.18)	0.2615
Comorbidity						
≥1	82 (1.9)	1.00 (ref)		27 (0.76)	1.00 (ref)	
<1	375 (1.4)	0.94 (0.74–1.20)	0.6333	193 (0.83)	0.81 (0.54–1.22)	0.3196

***Abbreviations***: DM, type 2 diabetes mellitus; HR, hazard ratio; CI, confidence interval.

a Number of subjects with colon cancer over total number of subjects in each group.

**Table 3:** Colon cancer risk in men before and after DM onset.

	Before DM Onset			After DM Onset		
	N (%)^a^	HR (95% CI)	*P*-value	N (%)^a^	HR (95% CI)	*P*-value
Diabetes status						
Yes	117 (2.0)	1.28 (1.04–1.58)	0.0223	56 (1.1)	1.18 (0.86–1.62)	0.3004
No	471 (1.7)	1.00 (ref)		171 (0.72)	1.00 (ref)	
Smoking						
Yes	484 (1.9)	1.00 (ref)		179 (0.83)	1.00 (ref)	
No	104 (1.3)	0.90 (0.72–1.11)	0.3137	48 (0.69)	0.77 (0.56–1.07)	0.1157
MESA residency						
Yes	95 (1.5)	1.00 (ref)		53 (0.95)	1.00 (ref)	
No	493 (1.8)	0.80 (0.64–1.00)	0.0496	174 (0.75)	1.14 (0.83–1.56)	0.4148
Birth year						
1929 and prior	277 (3.6)	36.2 (11.5–113.7)	<0.0001	85 (1.6)	43.1 (5.93–314.0)	0.0002
1930–1939	195 (2.3)	21.7 (6.92–68.1)	<0.0001	81 (1.2)	23.7 (3.28–171.2)	0.0017
1940–1949	90 (1.0)	9.39 (2.97–29.7)	0.0001	45 (0.57)	11.7 (1.61–85.0)	0.0152
1950–1959	23 (0.38)	3.46 (1.04–11.5)	0.0431	15 (0.26)	5.8 (0.76–43.8)	0.0893
1960 and later	3 (0.11)	1.00 (ref)		1 (0.04)	1.00 (ref)	
DM diagnosis period						
1995–1999	164 (2.2)	0.78 (0.62–1.00)	0.0511	88 (1.4)	0.80 (0.52–1.26)	0.3367
2000–2004	255 (1.8)	0.94 (0.77–1.14)	0.5156	99 (0.83)	0.89 (0.61–1.32)	0.5676
2005–2009	169 (1.4)	1.00 (ref)		40 (0.38)	1.00 (ref)	
BMI (kg/m^2^)						
<25	70 (2.0)	1.00 (ref)		23 (0.80)	1.00 (ref)	
25–29.9	392 (2.0)	0.92 (0.71–1.19)	0.5073	130 (0.78)	0.85 (0.54–1.33)	0.4692
≥30	126 (1.2)	0.78 (0.58–1.05)	0.1023	74 (0.81)	1.12 (0.70–1.82)	0.6439
Insurance						
Yes	454 (1.8)	1.00 (ref)		168 (0.79)	1.00 (ref)	
No	134 (1.6)	0.71 (0.57–0.88)	0.0014	59 (0.80)	0.90 (0.63–1.27)	0.5367
Comorbidity						
≥1	129 (2.5)	1.00 (ref)		30 (0.78)	1.00 (ref)	
<1	459 (1.6)	0.99 (0.81–1.21)	0.9036	197 (0.80)	0.81 (0.55–1.21)	0.3033

***Abbreviations***: DM, type 2 diabetes mellitus; HR, hazard ratio; CI, confidence interval; MESA, Marshfield Epidemiologic Survey Area; BMI, body mass index.

a Number of subjects with colon cancer over total number of subjects in each group.

**Table 4:** Number needed to be exposed to DM for one additional person to develop colon cancer.

Time (years)^a^	HR^b^	Survivalprobability (S_c_)^c^	NNEH^d^
**Women**			
Before DM Onset	1.03		
−15		0.9995	76942
−10		0.9986	27492
−5		0.9973	14264
−3		0.9962	10141
0		0.9938	6223
After DM Onset	1.30		
3		0.9956	759
5		0.9930	478
10		0.9863	245
13		0.9806	174
**Men**			
Before DM Onset	1.28		
−15		0.9989	3284
−10		0.9976	1506
−5		0.9943	635
−3		0.9922	465
0		0.9862	263
After DM Onset	1.18		
3		0.9972	1965
5		0.9954	1197
10		0.9904	575
13		0.9861	398

a Time 0 represents the date of DM onset/reference date.

b Hazard ratio of colon cancer in diabetic compared to non-diabetic subjects.

c The probability of a non-diabetic subject being alive and cancer-free at specified time.

d NNEH, number need to be exposed to DM for one additional person to be harmed (i.e. develop colon cancer).

Crude colon cancer incidence was higher after the reference date reaching 149.0 per 100 000 person-years in diabetic subjects and 117.6 per 100 000 person-years in non-diabetic subjects. Gender differences were again observed after the reference date, but there was no significant increase in colon cancer risk associated with DM in either men or women (Figure 4C and D). The trend toward increased risk of colon cancer in diabetic women after DM onset was insignificant (HR = 1.30, 95% CI 0.94–1.81, *P* = 0.1162). The HR for diabetic men decreased slightly from that before the reference date, but was also not significant at 1.18 (95% CI 0.86–1.62, *P* = 0.3004). Adjustments for comorbidities and insurance status had little effect on HR estimates before or after the reference date.

## Discussion

Several important physiological changes occur before overt clinical onset of DM (Figure 1). The insulin resistance and hyperinsulinemia characteristic of pre-diabetes may affect colon cancer risk before DM diagnosis, but epidemiological data regarding cancer incidence in the pre-diabetic state remain limited. In 2007, Giovannucci and Michaud [11] hypothesized that hyperinsulinemia is the critical factor responsible for increased colon cancer risk in DM, citing evidence from animal modeling and epidemiological studies [12]–[16]. However, this is the first report to comprehensively examine the temporal relationship between DM and colon cancer, including the time period before clinical DM onset, and the evidence presented here suggests that the greatest increase in the risk of colon cancer occurs before DM onset and is essentially limited to men.

Decreased NNEH over time to DM onset in men suggests a cumulative oncogenic effect with progression through the pre-diabetes phase, whereby fewer men need to be exposed to the pre-DM milieu over time for each additional case of colon cancer. No such difference was observed after clinical DM onset. Together, these findings support the notion that hyperinsulinemia and factors that cause hyperinsulinemia, such as obesity, physical inactivity, and an unbalanced diet, may result in increased oncogenic potential in the colon [17]. Lack of such an association in women suggests that gender differences in colon cancer risk are discernible early in the diabetes trajectory.

Several meta-analyses have been published that report a similar increase in risk of colon cancer in men or women with diabetes or metabolic syndrome [2], [6], [18]–[22]. However, there is also significant evidence that points to increase risk in men. In a 2009 systematic review and meta-analysis, Nguyen et al. [5] observed a predilection of colorectal adenomas for men (RR = 1.83, 95% CI 1.69–1.97). Further, a meta-analysis of screening colonoscopy studies reported a lower adenoma detection rate in trials enrolling predominantly women [23]. Consistent with our findings of a trend toward increased risk in women after the reference date, a large population-based, cross-sectional study found rates of colorectal neoplasia in women to reach rates of those in men approximately 10 years later in life [24]. A recent, large meta-analysis of 24 cohort studies, Luo et al. [4] found significant heterogeneity by gender and noted a stronger association between DM and colon cancer among males (RR = 1.47, 95% CI 1.15–1.86) than females (RR = 1.08, 95% CI 1.00–1.17). Similarly, two meta-analyses that examined the association between BMI, which is strongly related to DM and colon cancer risk by gender, found a greater association in males than females [25], [26]. While it seems that higher quality evidence largely suggests that colon cancer risk is greater in men than in women, we suspect that lack of observation of gender effects in some meta-analyses and reports, despite a clear effect of DM, could be related to lack of examination of the temporal changes in risk we report here, in addition to other difficulties in accurately determining date of DM onset and, quality of the reported data (e.g. reliance on self-reports).

Gender differences in colon cancer risk suggest the potential for hormonal effects. Obesity is the most common co-morbidity of DM and is implicated in promotion of oncogenic processes [27]. In the present study, diabetic subjects had a higher mean BMI at the reference date than those in the non-diabetic group. Obesity may differentially affect hormone levels in men and women, especially by reducing androgen levels in men. Obesity is a well-known risk factor for colorectal cancer [11], and lower androgen levels may also increase colorectal cancer risk in men [28]. The increased colon cancer risk observed before DM onset in men, but not women, may be related to this physiological mechanism. Several studies also suggest that female sex hormones may be protective for colon cancer [29]–[31]. Before menopause, hormonal cycling may delay the increase in risk for colon cancer noted in the present study among women with emergent DM. However, with advancing age, onset of menopause, and increased duration of DM, any advantages afforded by the female hormonal milieu may dissipate [32]. While the increased colon cancer risk in men before DM onset may appear relatively modest, the societal and economic impact of these findings is amplified in the context of the pervasiveness of DM. An estimated 79 million people in the United States meet criteria for pre-diabetes, with over one million cases of DM diagnosed annually [33]. The NNEH for colon cancer in diabetic men at time of DM onset is 263, suggesting that the pre-diabetic state is a risk factor for colon cancer on par with smoking for bladder cancer, where the NNEH is 727 [34]. Recognition of the increased risk for colon cancer in men before DM onset allows for potential intervention in the pre-diabetes state. Understanding the gender differences in colon cancer risk identifies the patients in which intervention during the pre-diabetes phase may have the greatest impact. Interventions such as weight control through diet and exercise may have a far reaching impact on the likelihood of colon cancer in pre-diabetic men.

The observational design of the present study results in the potential for certain types of bias. First, the percentage of diabetic subjects with 20 or more healthcare visits in the 2 years before the reference date was greater than that of non-diabetic subjects. Increased healthcare utilization by diabetic subjects raises the possibility of ascertainment bias. However, evidence indicates that there is no increased diligence in colon cancer screening in patients with emergent DM [35], [36]. Despite reports that diabetic individuals have higher rates of adenoma earlier in life, systematic early screening for diabetic patients is not currently recommended [37]. Second, data were collected during routine clinical care and not in the context of a systematic research study with baseline patient information collected only at reference date. Thus, there is a possibility that patient covariates may have been impacted by outcome. This, however, would be expected to bias results towards the null rather than produce spurious associations. Third, we also note that cancer treatment and screening data were unavailable for the majority of patients. Fourth, we focused our analyses on colon cancer to avoid confounding as a result of the physiological differences in tumorigenesis in the colon and rectum, which may limit comparison of our findings to previous studies of colorectal cancer. Finally, the potential for immortal time bias needs to be acknowledged, as patients destined to develop DM could have developed rapidly progressing colon cancer and died before DM onset could be observed. Bias is introduced when such patients are misclassified into the “unexposed” group or are excluded from analyses. However, the impact of this sort of bias is likely to move the pre-diabetic effect observed toward null, and in such a case, men may be at even greater risk for colon cancer during the pre-diabetes phase than demonstrated here. On the other hand, this study also has several strengths. We capitalized on a unique institutional capacity for tracking individual patient data over time to accurately define DM onset and colon cancer risk using a complex algorithm and comprehensive administrative and clinical parameters, including laboratory measures. Use of clinical parameters is unique to the present study. Given the insidious onset of DM, our ability to track clinical DM diagnosis within the context of elements in the EMR and laboratory and administrative data was a major strength of the current study.

### Conclusion

By accurately pinpointing date of clinical DM onset, we were able to assess the temporal relationship between DM and colon cancer risk and carefully analyze gender differences. We found that colon cancer risk is increased in diabetic men, but not women, before DM onset. Following DM onset, there was no association between DM and colon cancer risk in either gender. In pre-diabetic men, colon cancer risk increased over time, peaking near the time of DM onset, suggesting that the effects of the pre-diabetic state on colon cancer in men are cumulative. These results support the hyperinsulinemia hypothesis of carcinogenesis [11], [17]. It would be important to confirm these results in a well-conducted, prospective study, as the outcomes we report demonstrate an important temporal perspective regarding colon cancer risk and DM trajectory that has not been previously reported. If confirmed, the pre-diabetes phase may offer the greatest opportunity to implement an interventional or screening strategy to reduce risk of colon cancer in men.
